# Demographic and socioeconomic predictors of religious/spiritual beliefs and behaviours in a prospective cohort study (ALSPAC) in Southwest England: Results from the offspring generation

**DOI:** 10.12688/wellcomeopenres.18517.1

**Published:** 2022-12-05

**Authors:** Daniel Major-Smith, Jimmy Morgan, Isaac Halstead, Hamid Reza Tohidinik, Neil Goulding, Yasmin Iles-Caven, Jean Golding, Kate Northstone

**Affiliations:** 1 Centre for Academic Child Health, Population Health Sciences, Bristol Medical School, University of Bristol, Bristol, BS8 2BN, UK; 2 MRC Integrative Epidemiology Unit, University of Bristol, Bristol, UK; 3 Population Health Sciences, Bristol Medical School, University of Bristol, Bristol, BS8 2BN, UK

**Keywords:** ALSPAC, religion, confounding, bias, socioeconomic position, descriptive study.

## Abstract

*Background*: We explored associations between possible demographic and socioeconomic causes of religious/spiritual beliefs and behaviours (RSBB) in the offspring generation of the Avon Longitudinal Study of Parents and Children (ALSPAC).

*Methods:* We examined approximately 4,450 offspring aged 28 years with RSBB data from a prospective birth cohort study (ALSPAC) in Southwest England. Three RSBB outcome measures were assessed: religious belief (belief in God/a divine power; yes/not sure/no), religious affiliation (Christian/none/other) and religious attendance (frequency of attendance at a place of worship). We explored age- and sex-adjusted associations between 35 demographic and socioeconomic exposures and each of the three RSBB outcomes using multinomial regression. Exposure-sex interactions were also examined.

*Results*: Some sociodemographic factors were associated with RSBB in this cohort; for instance, being female and from an ethnicity other than White were associated with increased religiosity across all domains. For many other exposures, however, associations were frequently null or inconsistent, often depending on the specific exposure and outcome combination. As an example, higher educational attainment was associated with higher rates of religious attendance, but not with religious belief or affiliation; in contrast, higher income was associated with lower levels of religiosity. No consistent interactions between sex and the exposures on RSBB were found. Effect sizes were also rather weak, with most pseudo- *R* ^2^ values below 0.5% and a maximum of 1.2%.

*Conclusions*: The results highlight that several demographic and socioeconomic factors are associated with RSBB in this cohort. However, the number of these associations, and their magnitude, is smaller than comparable results from the parental generation of these offspring, suggesting that patterns of sociodemographic factors associated with RSBB differ between these generations. In addition to describing these associations, this paper will help inform future studies using these data, particularly regarding the choice of potential sociodemographic confounders.

## Introduction

A growing body of research has shown that religious/spiritual beliefs and behaviours (RSBB) are associated with various health outcomes, such as improved mental health and lower rates of overall mortality ^1, 2^. While randomised-controlled trials assessing the effects of RSBB on health are practically impossible, studies using longitudinal data suggest that some of these associations may be causal ^3, 4^. Nonetheless, longitudinal data on both RSBB and health from population-based studies using prospective data collection, large sample sizes and detailed data on potential confounders are rare, which may explain why RSBB is often overlooked in the majority of health research ^5^.

A further difficulty regarding research into RSBB and health, especially when the research aim is causal inference ^6– 8^, is identifying appropriate covariates in order to remove bias due to confounding. This is difficult for two reasons.

First, it is not clear which variables cause RSBB, and hence may be confounders of the RSBB-health relationship. Although many factors have been proposed which may cause RSBB – subsumed under the three broad categories of ‘socioeconomic’, ‘cognition/psychology’ and ‘cultural transmission’ ^9, 10^ – research assessing whether these associations reflect causal relations is generally lacking, relatively weak or contradictory ^9, 11^. For instance, lower levels of educational attainment – often used as a proxy for socio-economic position (SEP) – have been associated with higher religiosity ^12, 13^, lower religiosity ^14^, no association with religiosity ^9, 15^, and perhaps more complex associations depending on religious denomination and the measure of RSBB ^16^. Whether SEP causes RSBB may therefore depend on many context-specific factors, such as country-level differences (much of the previous research has been conducted in the United States), the specific aspect of SEP, and the measure of RSBB used.

The second difficulty of identifying confounders of the RSBB-health relationship is that, even for variables which may plausibly cause RSBB, there is the potential that many of these are also *caused by* RSBB; that is, *bidirectional causation*. For instance, marital status may cause RSBB (as marriage may result in an increase in religious belief/attendance), but RSBB may also cause marital status (as religious individuals may be more likely to get and remain married). In other words, many factors may be both confounders and mediators of the RSBB-health relationship ^2, 14^. In such situations of bidirectional causation – and in the absence of longitudinal data – it is not possible to appropriately adjust for confounding.

Despite these difficulties, identifying potential confounders is necessary to reduce bias when estimating causal effects. In this paper, we examine whether a wide range of demographic and socioeconomic factors are associated with RSBB in the offspring from a prospective birth cohort (the Avon Longitudinal Study of Parents and Children; ALSPAC). We focus on demographic and socioeconomic variables here because these factors are hypothesised to cause RSBB ^9, 10, 13, 17, 18^ and are known to impact health ^19^, meaning they are likely to be included as confounders in many analyses exploring the RSBB-health relationship. While we are not making causal claims in this paper, exploring associations between sociodemographic variables and RSBB will help inform the choice of potential confounders in future studies. In addition to this aim, simply describing these associations will be of use in understanding the patterns of results in this cohort, and permit comparisons to results from other studies to assess whether these associations differ. A previous paper explored associations between sociodemographic factors and RSBB in the parental generation of ALSPAC ^14^; the present paper explores these associations in the offspring generation of this cohort, and examines any differences in the patterns of association between the two generations.

## Methods

### Participants

Pregnant women resident in Bristol (UK) and surrounding areas with expected dates of delivery between 1 ^st^ April 1991 and 31 ^st^ December 1992 were invited to take part in the study. The initial number of pregnancies enrolled was 14,541, of which there were a total of 14,676 foetuses, resulting in 14,062 live births and 13,988 children who were alive at one year of age ^20, 21^. When the oldest children were approximately seven years of age, an attempt was made to bolster the initial sample with eligible cases who had failed to join the study originally, resulting in an additional 913 children being enrolled. The total sample size for analyses using any data collected after the age of seven is therefore 15,447 pregnancies, resulting in 15,589 foetuses; of these, 14,901 were alive at one year of age ^22^.

The current research focuses specifically on the offspring generation (known as either ‘G1’ [ALSPAC Generation-1] or ‘YPs’ [ALSPAC Young People]). After removing observations for participants who had withdrawn consent for their data to be used, a total of 14,843 YPs were included in the final dataset (although only approximately 4,450 of these had RSBB data; see below). Study data gathered since the YPs were aged 22 were collected and managed using REDCap electronic data capture tools hosted at the University of Bristol ^23^. REDCap (Research Electronic Data Capture) is a secure, web-based software platform designed to support data capture for research studies. Please note that the study website contains details of all the data that is available through a fully searchable data dictionary and variable search tool: http://www.bristol.ac.uk/alspac/researchers/our-data/.

### Outcome measures

The outcome variables for this study were the YPs’ RSBB ( Table 1), measured in late 2019 to early 2020 when the index offspring were approximately 28 years of age (mean = 27.9; SD = 0.51; range = 26.8 to 29.2). Although various RSBB measures were assessed at this time-point ^24^, we consider three which encompass a range of religious beliefs and behaviours and enable comparability with the RSBB outcomes used in the previous paper on ALSPAC parents ^14^: religious belief (belief in God or some divine power; yes vs not sure vs no); religious affiliation (Christian vs none vs other); and religious attendance (frequency of attendance at a place of worship; at least once a month vs at least once a year vs occasionally vs not at all). Religious belief and religious affiliation were coded the same for the index offspring as they were for parents, but for religious attendance the categories were slightly different. Due to the small numbers of YPs answering ‘at least once a week’ or ‘at least once a month’, these responses were combined (while they were left separate for parents). Additionally, YPs had an ‘occasionally’ option, which was not available for parents when measured in pregnancy. As this questionnaire was administered nearly 30 years after initial ALSPAC enrolment in pregnancy, missing data is quite substantial, with 70% of the 14,843 offspring having missing RSBB data ( Table 1).

**Table 1.** Summary of religious/spiritual beliefs and behaviours (RSBB) outcomes used in this study for ALSPAC offspring when aged approximately 28 years ( *n* = 14,843).

RSBB outcome	Response	N (%)
**Belief in God/a divine power**	*Yes*	756 (17.0%)
	*Not sure*	1,195 (26.8%)
	*No*	2,505 (56.2%)
	*Total*	4,456
Missing data		10,387 (70.0%)
**Religious affiliation**	*None*	2,890 (65.4%)
	*Christian*	1,220 (27.6%)
	*Other*	309 (7.0%)
	*Total*	4,419
Missing data		10,424 (70.2%)
**Frequency of attendance at** **a church/place of worship**	*At least once a month ^a^*	204 (4.6%)
	*At least once a year*	340 (7.7%)
	*Occasionally*	588 (13.3%)
	*Not at all*	3,287 (74.4%)
	*Total*	4,419
Missing data		10,424 (70.2%)

^a^ This category includes participants who responded both ‘at least once a week’ ( *n* = 133) and ‘at least once a month’ ( *n* = 71).

### Exposure measures

We examined a range of demographic and socioeconomic exposures which may potentially cause RSBB. This includes a number of SEP-related variables, such as educational attainment, income, occupational social class, employment, home ownership status, recent financial difficulties and area-level deprivation, measured in the offspring and/or their parents. A summary of these sociodemographic variables is given in Table 2, with full descriptive statistics provided in Table S1 (please see Extended data for supplementary tables and figures ^25^). All parental exposures were measured in pregnancy or shortly afterwards, while YP exposures were measured at the same time as the RSBB questions or shortly before (up to two years prior). We explored both parental and offspring exposures to examine how different sociodemographic factors throughout the lifespan are associated with RSBB.

**Table 2.** Summary of demographic and socioeconomic variables used as exposures.

Variable ( *variable name*)	Variable coding	When measured
*Demographic variables*		
Age ( *AgeAt28*)	Continuous (months)	Approx. age 28 years
Mother’s age at birth of study child ( *mother_ageAtBirth*)	Continuous (years)	At birth
Sex ( *male*)	Binary (Female vs Male)	At birth
Ethnicity ( *nonWhiteEthnic*)	Binary (White vs Other than White)	In pregnancy (with recent info to fill in missing data)
Lives with a partner ( *livePartner*)	Binary (No vs Yes)	Approx. age 28 years
Mother’s marital status ( *maritalStatus_* *mat*)	Unordered category (never married vs currently married vs widowed/divorced/separated)	In pregnancy
Mother’s residential mobility (in last 5 years; *mobility_mat*)	Ordered category (0 moves vs 1 move vs 2 moves vs 3 moves vs 4 moves vs 5 or more moves)	In pregnancy
Urban/rural status ( *rural*)	Binary (town/village/hamlet vs urban)	January 2020 (approx. age 28 years)
Mother’s urban/rural status ( *rural_mat*)	Binary (town/village/hamlet vs urban)	January 1993 (approx. birth)
Is a parent ( *parent*)	Binary (No vs Yes)	Approx. ages 20 to 28 years
Mother’s parity at birth ( *parity_mat*)	Ordered category (0 vs 1 vs 2 or more)	In pregnancy
*Socioeconomic variables*		
Highest education qualification ( *education*)	Ordered category (GCSE/none vs vocational vs A-level vs degree) ^a^	Approx. age 27 years
Mother’s highest education qualification ( *education_mat*)	Ordered category (CSE/none vs vocational vs O-level vs A-level vs degree) ^a^	In pregnancy
Father’s highest education qualification ( *education_pat*)	Ordered category (CSE/none vs vocational vs O-level vs A-level vs degree) ^a^	In pregnancy
Currently employed ( *employed*)	Binary (No vs Yes)	Approx. age 28 years
Mother’s occupational social class ( *highSocClass_mat*)	Binary (low [III manual/IV/V] vs high [I/II/III non-manual]) ^b^	In pregnancy
Father’s occupational social class ( *highSocClass_pat*)	Binary (low [III manual/IV/V] vs high [I/II/III non-manual]) ^b^	In pregnancy
Low social class in childhood ( *lowSocClass_0_16*) ^c^	Binary (No vs Yes)	Pregnancy to age 16 years
Monthly income ( *income*)	Ordered category (£0-£499 vs £500-£999 vs £1000-£1499 vs £1500-£1999 vs £2000 and above)	Approx. age 26 years
Parental weekly household income ( *income_parents*)	Continuous (log income per/week)	When study child was approx. aged 3/4 years
Index of multiple deprivation ( *IMD*)	Ordered category (1 ^st^ quintile [least deprived] vs 2 ^nd^ quintile vs 3 ^rd^ quintile vs 4 ^th^ quintile vs 5 ^th^ quintile [most deprived])	January 2020 (approx. age 28 years)
Mother’s index of multiple deprivation ( *IMD_mat*)	Ordered category (1 ^st^ quintile [least deprived] vs 2 ^nd^ quintile vs 3 ^rd^ quintile vs 4 ^th^ quintile vs 5 ^th^ quintile [most deprived])	January 1993 (approx. birth)
Townsend deprivation index ( *townsendDep*)	Ordered category (1 ^st^ quintile [least deprived] vs 2 ^nd^ quintile vs 3 ^rd^ quintile vs 4 ^th^ quintile vs 5 ^th^ quintile [most deprived])	January 2020 (approx. age 28 years)
Mother’s Townsend deprivation index ( *townsendDep_mat*)	Ordered category (1 ^st^ quintile [least deprived] vs 2 ^nd^ quintile vs 3 ^rd^ quintile vs 4 ^th^ quintile vs 5 ^th^ quintile [most deprived])	January 1993 (approx. birth)
Housing status ( *housing*)	Unordered category (owned/mortgaged vs renting vs council/housing association vs other)	Approx. age 28 years
Mother’s housing status ( *housing_mat*)	Unordered category (owned/mortgaged vs renting vs council/housing association vs other)	In pregnancy
Recent financial difficulties ( *financeDiffs*)	Binary (No vs Yes)	Approx. age 22 to 27 years
Family financial difficulties in childhood ( *financeDiffs_0_16*) ^c^	Binary (No vs Yes)	Pregnancy to age 16 years
Mother’s financial difficulties score ( *financeDiffsScore_mat*)	Continuous (from 0 [no difficulties] to 15 [severe difficulties])	In pregnancy
Parental access to car ( *accessToCar_* *parents*)	Binary (No vs Yes)	In pregnancy
Crowding index ( *crowding_birth*)	Ordered category (calculated by dividing the number of people in the household by the number of rooms; ≤ 0.5; > 0.5 to 0.75; > 0.75 to 1; > 1)	In pregnancy
Bad neighbourhood in childhood ( *badNeigh_0_16*) ^c^	Binary (No vs Yes)	Pregnancy to age 16 years
Mother’s self-reported neighbourhood quality index ( *neighPercept_mat*)	Continuous (score from 0 [low quality neighbourhood] to 12 [high quality neighbourhood])	In pregnancy
Father absence in childhood ( *fatherAbsence*)	Binary (father present vs father absent)	Pregnancy to age 18 years
Age of father absence ( *age_* *fatherAbsence*)	Unordered category (No father absence vs father absence age 0 to 4 years vs father absence age 5 or older)	Pregnancy to age 18 years

^a^ GCSE = General Certificate of Secondary Education qualification (compulsory examinations sat at the end of secondary school at approx. age 16; introduced in 1986 to replace CSE and O-levels); CSE = Certificate of Secondary Education qualification (examinations sat at the end of secondary school at approx. age 16; compulsory from the early 1970s, unless completing O-level qualifications instead; replaced in 1986 by GCSEs); O-level = Ordinary level qualifications (examinations sat at the end of secondary school, often for more academically-able pupils at approx. age 16; replaced in 1986 by GCSEs); A-level = Advanced level qualification (non-compulsory examinations sat at the end of college or sixth form at approx. age 18). ^b^ For more information on these occupation social classes, see: https://sru.soc.surrey.ac.uk/SRU9.html. Note also that these occupational social class variables were not available for the ALSPAC offspring generation. ^c^ For more information on how these variables summarising exposures from birth to age 16 years were coded and the specific variables used, see 26.

### Confounder variables

As this paper simply aims to describe the broad associations of sociodemographic factors which may plausibly cause RSBB analyses here will only adjust for age and sex (other than the age-only and sex-only models). Further research is necessary to make causal claims, but adjusting for age and sex removes two common sources of confounding.

### Analysis

We first explored the correlations between each of the exposure variables to examine relations between these variables, with a strong correlation potentially indicating that they measure similar constructs. Continuous, ordered categorical and binary variables were assessed using Spearman's correlation. As correlation coefficients for unordered categorical variables (mother’s marital status, and YP’s and mother’s home ownership status) could not be estimated in this way, they were approximated via multinomial regression with these variables as the outcome and then square-rooting the pseudo- *R ^2^* value (cf. ^27^).

Next, we explored associations between each of the sociodemographic exposures ( Table 2) and each of the RSBB outcomes ( Table 1). As religious belief and affiliation are unordered categorical variables, multinomial regression was used. We also analysed frequency of religious attendance using multinomial regression because: i) the proportional odds assumption was violated when running an ordinal regression model (tested via a Brant test; multinomial models do not require this assumption); and ii) using multinomial models for each RSBB outcomes means the coefficients are on the same scale (relative risk ratios), facilitating interpretation and comparison of effect sizes between these outcomes. Other than the age-only and sex-only models, all models were adjusted for both age and sex. As religiosity is generally known to be higher in females, both cross-culturally ^28^ and as reported in ALSPAC specifically ^24^, we examined whether these sociodemographic exposures differed by sex, by including an interaction between sex and each exposure. Given that all YPs were approximately the same age (28 years), we did not explore interactions between age and the exposures.

For each exposure-outcome combination we performed two likelihood ratio tests to assess model fit. The first tested whether adding the exposure to the model resulted in an increase in model fit, relative to the baseline age- and sex-only model (or an age-only model when sex was the exposure, or a sex-only model when age was the exposure). The second tested whether the addition of an interaction term between sex and the exposure improved model fit, relative to the model without this interaction term. In an attempt to minimise the false discovery rate, we used a Bonferroni-correction based on the number of exposures tested; as there were 35 exposures, this gave a Bonferroni-corrected threshold when using a traditional 0.05 alpha value of 0.05/35 = 0.0014 (0.04/34 = 0.0015 for the interaction models). Rather than arbitrarily dichotomise findings into ‘significant’ and ‘non-significant’, these adjusted *p*-value thresholds are instead intended as an informative summary statistic to describe large numbers of associations and assess the strength of evidence against the null hypothesis of there being no association between the exposure and outcome ^29^. To provide a measure of effect size, we used the difference in McFadden’s pseudo- *R ^2^* value ^30^ between the model with vs without the exposure (or with vs without the interaction term, for interaction models). While this pseudo- *R ^2^* value is not completely analogous to a standard *R ^2^* ‘variance explained’ value for linear models ^31^, it can be a useful statistic for assessing model fit and comparing between exposures. Analyses were conducted in Stata v.17 but can also be performed in R (R Project for Statistical Computing), an open-source alternative.

## Results

Descriptive statistics for each RSBB outcome are presented in Table 1. The majority of YPs did not believe in God/a divine power (56%) and only 17% were definite believers. Correspondingly, two-thirds of participants did not report a religious affiliation, while 28% identified as Christian (of various denominations, but predominantly Church of England/Protestant) and 7% as another religion (small numbers of Buddhist, Sikh, Hindu and Muslim, but largely made up of ‘other’ responses). Frequency of attendance at a place of worship was also low, with only 5% of YPs attending at least once a month and 8% attending at least once a year; 13% attended occasionally, while three-quarters did not attend at all. Descriptive statistics for each exposure are displayed in the Extended data, Table S1, while descriptive statistics for these exposures, split by each of the RSBB outcome categories, are displayed in Tables S2 (for religious belief), S3 (for religious affiliation) and S4 (for religious attendance) ^25^.

**Figure 1.**
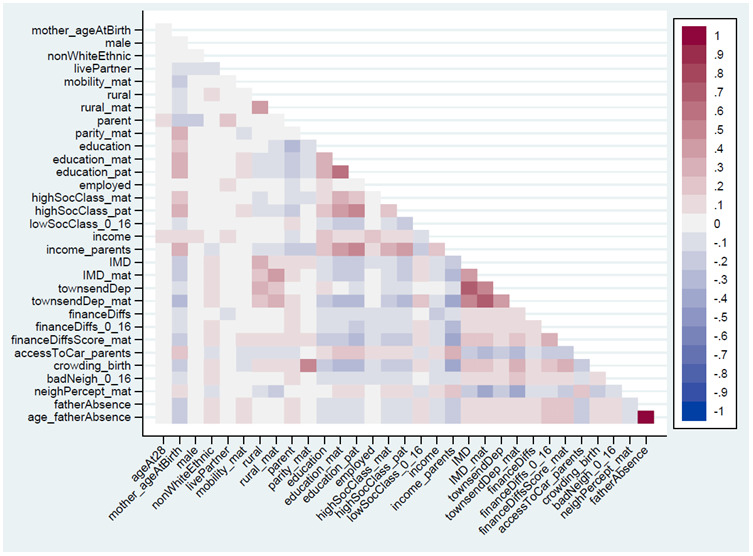
Heat-plot of the Spearman’s correlation matrix between all continuous, ordered categorical and binary exposures used in analysis (numerical results are displayed in Table S5 ^25^). For full details on the variables included here, see Table 2.

The Spearman’s correlations between the 32 continuous, ordered categorical and binary exposure variables are summarised in Figure 1 (see Table S5 for the full correlation coefficients; pseudo-correlation coefficients for the unordered categorical variables mother’s marital status, home ownership status and mother’s home ownership status are in Table S6 ^25^). Overall, other than some clusters measuring similar variables – such as Index of Multiple Deprivation (IMD) and Townsend Deprivation Index, or the two measures of father absence – correlations between these different exposures were generally quite weak, even for similar variables measured in both generations. For instance, the correlation between maternal and offspring education was 0.32, while the correlation between parental and offspring income was 0.19. Although many exposures are somewhat correlated, the relatively small coefficients between these variables indicates that they are to some extent independent and measure somewhat different aspects of sociodemographic circumstances.

**Figure 2.**
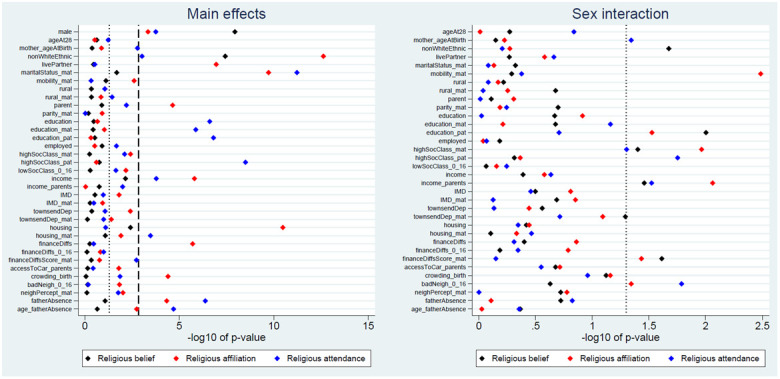
*P*-values for each exposure and RSBB outcome. The left-hand plot shows the age- and sex-adjusted main effects; the right-hand plot shows the interaction between sex and the exposure. The light dashed line indicates a standard 0.05 *p*-value threshold; the thicker dashed line denotes the Bonferroni-corrected *p*-value threshold (0.05/35 = 0.0014 for main effects and 0.05/34 = 0.0015 for interaction effects). Results to the right of these lines indicate a *p*-value below said threshold. For full details on the variables included here, see Table 2. For sample sizes, see Tables S9–S11.

We next explored associations between each exposure and the three RSBB outcomes, with a summary of the *p*-values from these likelihood ratio tests in Figure 2. For religious belief, only 2 of 35 (6%) exposure main effects were associated with this outcome at the Bonferroni-adjusted level, while 5 (14%) met a standard 0.05 alpha threshold. More exposure main effects were associated with religious affiliation (10 [29%] using Bonferroni-correction; 21 [60%] at a traditional 0.05 threshold) and religious attendance (11 [31%] using Bonferroni-correction; 21 [60%] at a traditional 0.05 threshold). This suggests that associations between the exposures and RSBB differ depending on the facet of RSBB measured; this can be seen in Figure 2, where sex is more strongly associated with religious belief than affiliation or attendance, while education is associated with religious attendance but not belief or affiliation. None of the sex-interaction models reached the Bonferroni-adjusted threshold, although for each outcome 5 to 6 (15% to 18%) reached a standard 0.05 level. These results are summarised in Table 3, with full *p*-values from all likelihood ratio tests in Table S7 ^25^.

**Table 3.** Summary of associations between 35 exposures and the three RSBB outcomes at both the Bonferroni-corrected and standard 0.05 alpha levels. Results show the number (percentage) of exposures below both the Bonferroni-corrected and traditional 0.05 alpha levels for each RSBB outcome.

	Number (%) of main effects below *p*-value thresholds		Number (%) of interactions below *p*-value thresholds	
	Bonferroni-corrected (0.05/35 = 0.0014)	0.05	Bonferroni-corrected (0.05/34 = 0.0015)	0.05
Religious belief	2 (6%)	5 (14%)	0 (0%)	5 (15%)
Religious affiliation	10 (29%)	21 (60%)	0 (0%)	6 (18%)
Religious attendance	11 (31%)	21 (60%)	0 (0%)	5 (35%)

**Figure 3.**
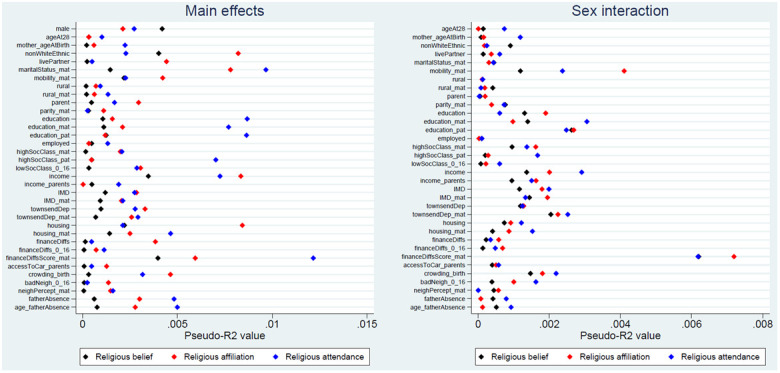
Pseudo- *R ^2^* values for each exposure and RSBB outcomes. The left-hand plot shows the age- and sex-adjusted main effects; the right-hand plot shows the interaction between sex and the exposure. For full details on the variables included here, see Table 2. For sample sizes, see Tables S9–S11.

The pseudo- *R ^2^* statistics for each of the exposure-outcome associations are presented in Figure 3 (with full results in Table S8 ^25^). Although these pseudo- *R ^2^* figures are not directly interpretable as measures of variance explained, in general these values are rather small, indicating that improvements in model fit associated with each exposure are relatively minor. The majority of pseudo- *R ^2^* values are below 0.5%, while the largest is only 1.2% (for maternal financial difficulties score and religious attendance). Pseudo- *R ^2^* figures are even smaller for the sex-interaction models, with most below 0.2%, and a maximum of only 0.7% (for maternal financial difficulties score and religious affiliation). Together, these likelihood ratio and pseudo- *R ^2^* results indicate that although a number of exposure main effects may have been associated with RSBB outcomes – especially for religious affiliation and religious attendance – the strength of the associations was relatively weak. It also appears that few of these exposure-outcome associations are moderated by sex.

The focus above on *p*-values and pseudo- *R ^2^* statistics can inform us about whether an association exists between an exposure and outcome, and the strength of this association, but does not provide information about the direction of said association; we therefore next focus on model parameter estimates to examine the direction and magnitude of these associations. As there are a large number of potential exposure-outcome combinations, here we will only describe a few key results in detail, with full results given in Tables S9–S11 ^25^.

Taking demographic variables first, as previously reported ^24^ females displayed greater religiosity across all RSBB outcomes, especially for religious belief, although attendance at a place of worship at least once a year and at least once a month was similar between the sexes (Extended data, Figure S1 ^25^). To put these relative risk ratios in context, 15% of males believe in God/a divine power, compared to 18% of females, while 62% of males do not believe, compared to 53% for females (Table S2 ^25^). Ethnicity was also strongly associated with each of the RSBB outcomes, particularly religious belief and affiliation, with ethnicities other than White more likely to believe in God/a divine power, have an ‘other religion’ affiliation, and attend a place of worship more frequently (Figure S2 ^25^). YPs who lived with a partner were less likely to identify as an ‘other religion’, although no differences in religious belief or attendance were observed (Figure S3 ^25^). Regarding mother’s marital status, YPs whose mothers were married were more likely to attend church more regularly, have a Christian religious affiliation, and somewhat more likely to believe in God/a divine power, relative to YPs from mothers who were not married (Figure S4 ^25^). Being a parent was associated with slightly greater religious belief, an increased probability of having a Christian affiliation, and somewhat lower chances of religious attendance at least once a year (Figure S5 ^25^). The YP’s age, maternal age at birth of the study child, maternal residential mobility, urban/rural location (of YP and their mother) and maternal parity had little-to-no association with any of the RSBB outcomes examined.

**Figure 4.**
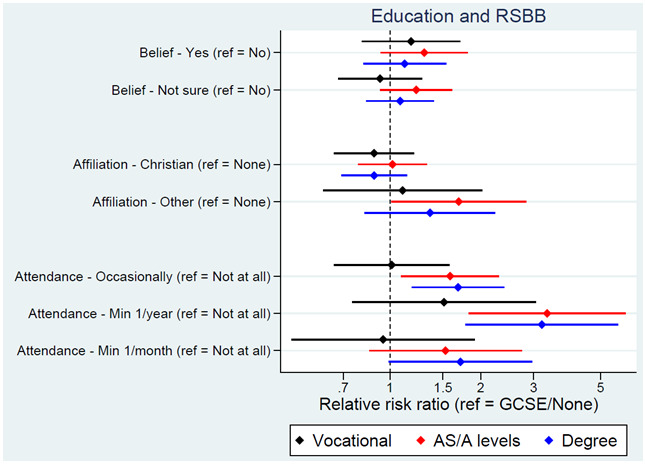
Associations between education and RSBB outcomes. All results are from multinomial regression analyses and show the relative risk ratio for a given educational level relative to both the educational reference level (GCSE/None) and the outcome reference (specified on the y-axis). Error bars are 95% confidence intervals. Sample sizes: religious belief = 3,281; religious affiliation = 3,253; religious attendance = 3,252.

We next turn to socioeconomic exposures. Educational attainment of the YPs, mothers and fathers was associated with religious attendance, but not religious belief or affiliation, with higher qualifications associated with a higher probability of attending a place of worship more regularly ( Figure 4 for YP education; Figure S6 for maternal education ^25^). Based on this model, 82% of YPs with GCSEs/no qualifications were predicted not to attend a place of worship, compared to 70% of those with a degree. The YP’s employment status, maternal and paternal social class, and low social class during childhood had little association with RSBB, although curiously higher paternal – but not maternal – social class was associated with attending a place of worship more regularly.

**Figure 5.**
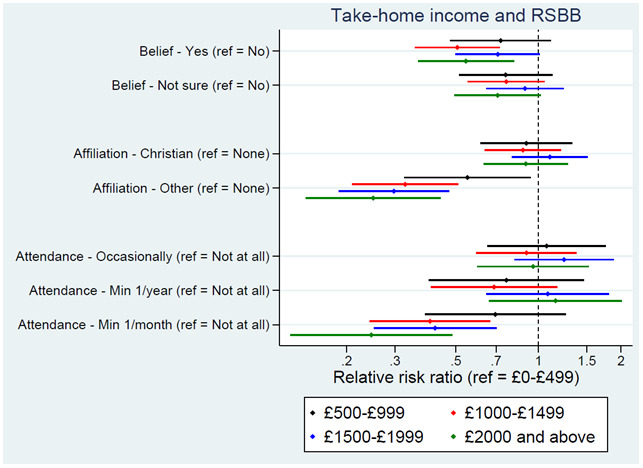
Associations between income and RSBB outcomes. All results are from multinomial regression analyses and show the relative risk ratio for a given income level relative to both the income reference level (£0-£499) and the outcome reference (specified on the y-axis). Error bars are 95% confidence intervals. Sample sizes: religious belief = 3,099; religious affiliation = 3,081; religious attendance = 3,072.

Parental income in early childhood had little association with RSBB, although the YP’s income was associated with both religious affiliation and attendance, and to a lesser extent belief; higher income was associated with somewhat lower rates of belief, less likely to identify as an ‘other religion’, and lower probabilities of regularly attending a place of worship ( Figure 5). Again, to provide some context, of those on the lowest income level (£0–£499 per month) 23% were predicted to answer ‘yes’, 28% ‘not sure’, and 48% ‘no’ for religious belief, compared to 16%, 25% and 60%, respectively, for those in the highest income level (£2000 and above). For religious affiliation, of those on the lowest income, 26% were predicted to be Christian, 15% to be another religion, and 59% no religion, compared to 28%, 4% and 68%, respectively, for those on the highest income. For religious attendance, of those on the lowest income, 69% were predicted to never attend, 13% to attend occasionally, 8% to attend at least once a year, and 10% to attend at least once a month, compared to 75%, 13%, 10% and 3%, respectively, for those on the highest income.

Relative to YPs who own their home, participants who rent or live in council/housing association accommodation were less likely to identify as Christian, while those who rent or live in other accommodation were more likely to have another religious affiliation; associations with religious belief were weaker, albeit with those renting and living in council/housing association being less likely to answer ‘not sure’, while results for religious attendance were largely null, but with somewhat lower rates of attendance if living in a council/housing association home (Figure S7 ^25^). There was little association between maternal home ownership status and RSBB, although YPs whose mothers lived in council/housing association accommodation were less likely to regularly attend a place of worship, while those with ‘other’ accommodations were more likely to attend.

Financial difficulties of the YP recently, during childhood, and of the mother in pregnancy, had little association with RSBB, although recent YP difficulties were associated with identifying as a non-Christian religion. Higher levels of household crowding at birth were associated with both lower rates of Christian affiliation, but higher rates of identifying with another religion; no strong associations between crowding and religious belief were reported, although increased crowding was somewhat associated with lower rates of religious attendance (Figure S8 ^25^). Father absence during childhood was associated with a lower likelihood of identifying as Christian, being less likely to attend a place of worship, and slightly lower rates of religious belief (Figure S9 ^25^). Area-level deprivation (as measured by either IMD or the Townsend index, in both YPs and mothers), parental access to a car, and perceptions of neighbourhood quality (both in pregnancy and throughout childhood) had little association with RSBB.

None of the interactions between the exposure and sex reached the Bonferroni-corrected threshold ( Figure 2). Given the number of associations explored, many of the results below than the traditional alpha level of 0.05 are therefore likely to be false positives. Given this, and the weak and inconsistent results of the sex-interaction models more generally ( Figure 3), we will not describe these interaction results further here (full results can be found in the Extended data, Tables S9–S11 ^25^).

## Discussion

This paper aimed to explore whether demographic and socioeconomic factors were associated with various aspects of RSBB in this cohort of young adults (aged approximately 28 years). The strongest and most consistent effects were seen with sex and ethnicity; females and those from ethnic backgrounds other than White displaying greater religiosity over the three RSBB domains assessed here (religious belief, affiliation and attendance). The majority of the other sociodemographic variables had either null or inconsistent associations with RSBB ( Figure 2); for instance, higher levels of education were associated with increased religious attendance, but not religious belief or affiliation, while being a parent, living with a partner and owning one’s home were associated with identifying as Christian, but less with religious belief or attendance. Overall, there were more associations with religious affiliation and attendance than there were for religious belief ( Table 3). Although numerous associations were found (as suggested by *p*-values), it is important to note that effect sizes were small, with most pseudo- *R ^2^* values for main effects below 0.5%, and a maximum of 1.2%, suggesting that these factors explain little of the variance in RSBB ( Figure 3). Interactions between sex and the exposures returned largely null results, with correspondingly small effect sizes. It is important not to interpret these results as causal estimates, but they will hopefully be of use when thinking about relationships between sociodemographic factors and RSBB, and will help inform the choice of potential sociodemographic confounders in future studies using this RSBB data.

One notable finding is that there is considerable heterogeneity in terms of both exposures and RSBB outcomes. That is, different aspects of socioeconomic background, although somewhat related, had varying associations with RSBB, such as education and income being associated with some aspects of RSBB, while measures of deprivation and employment had little relationship. Additionally, as noted above, associations between an exposure and one aspect of RSBB (say, religious attendance) did not always entail an association with another aspect of RSBB. This has two implications: i) different proxies for socioeconomic background may not be interchangeable, as their relationship with RSBB differs (e.g., adjusting for education may not be the same as adjusting for income, deprivation or occupational social class); and ii) RSBB outcomes are not interchangeable either, meaning that different measures of RSBB assess somewhat-independent facets of religiosity.

While acknowledging that these results only reflect descriptive associations, rather than causal estimates, comparisons to previous work may be useful, especially when compared against the recent ALSPAC paper looking at similar sociodemographic associations with RSBB in the parental generation ^14^. Some previous work, predominantly in North America, has found a negative relationship between various socioeconomic factors and religiosity, with less educated, and more deprived and marginalised individuals displaying increased religiosity ^13, 17, 32^. These patterns were somewhat supported among ALSPAC YPs, as higher income was associated with less frequent attendance at a place of worship and being less likely to identify with a non-Christian religion. However, we found little association between socioeconomic factors and religious belief more generally, while higher educational attainment was associated with increased religious attendance. These patterns differ from those of ALSPAC parents, where the majority of socioeconomic factors were positively associated with religious belief, affiliation and attendance (e.g., increased education, income, occupational social class, in addition to lower area-level deprivation, were associated with increased religiosity among ALSPAC parents). More associations with RSBB were found in the parent generation (compare Table 3 here to Table 3 in 14); in part this is likely due to larger sample size in parents (~12,000 for mothers vs ~9,500 for mothers’ partners vs ~4,400 for YPs), but effect sizes also appear to be larger in the parental generation as well. As such, associations between RSBB and sociodemographic variables appear to differ between the generations, both in magnitude and even in direction, as well as being generally much weaker in the offspring generation. Although the mean age of the parents was similar to the YPs here (approx. 28 years), direct comparisons may not be straightforward because the two populations are different; first, there is nearly a 30-year gap between the parental and YP measures of RSBB, and second, the parental generation were all parents (or parents-to-be), while the offspring generation includes all YPs, regardless of parental status. Nonetheless, these comparisons between the generations are informative, and highlight the changing nature of RSBB over time within this population.

### Strengths and limitations

One of the main strengths of this work is that it uses a large-scale, deeply-phenotyped, longitudinal population-based birth cohort with detailed information on both RSBB and potential sociodemographic variables which could be used as confounders in future studies. This level of detail, especially when combined with the intergenerational nature of ALSPAC (spanning both parental and offspring generations), is likely unparalleled amongst population-based studies, allowing an in-depth examination of the factors associated with different aspects of RSBB.

One of the key limitations of this study is the underlying assumption that these demographic and socioeconomic variables are *causes*, rather than *consequences*, of RSBB. That is, for these variables to remove bias due to confounding from future studies, these sociodemographic variables need to be causes of RSBB; if these variables are caused by RSBB (or causation is bidirectional), then these variables may also be mediators of the relationship between RSBB and some outcome of interest, meaning that adjusting for said covariate would bias the intended causal effect ( 6, 8, 33; for a more detailed discussion of this in relation to RSBB, see the discussion of 14). Some of the sociodemographic factors examined here do not suffer from this problem because they cannot be caused by RSBB, such as age, sex and ethnicity. For other factors measured in YPs this issue of reverse causation is difficult to overcome, especially as RSBB has so far only been measured once in this generation (although future RSBB questionnaires are planned). Parental sociodemographic factors also obviously cannot be caused by their offspring’s RSBB – removing the issue of reverse causality – although interpretation of these effects may not be straightforward either as these associations may be confounded by other factors such as parental RSBB, genetics, or other factors. Additionally, it is not clear whether parental socioeconomic variables are a valid proxy for the child’s socioeconomic position as an adult, especially given the rather low correlations between similar variables measured in both parents and children ( Figure 1). Longitudinal data on both RSBB and these covariates is needed to untangle these complex problems ^3^.

A further issue is the risk of selection bias, as only ~30% of enrolled ALSPAC offspring have RSBB data here (reduced somewhat in many of the analyses due to additional missing exposure data). If this missing data is related to both the outcome and the exposure, then this may result in selection bias, giving distorted estimates of the associations reported here ^6, 34– 36^. This may well be possible, as many of the exposures are known to be associated with continued ALSPAC participation, such as sex, socioeconomic position and ethnicity ^20, 21, 37^; additionally, the RSBB outcomes assessed here are also likely to be related to ALSPAC participation, with greater religiosity associated with increased study participation ^38^. The direction and magnitude of this potential bias depends on the strength and direction of selection in the exposure and outcome, so it is difficult to fully-anticipate the impact of potential selection bias. For instance, if both the exposure and outcome positively (or both negatively) predict selection, then the coefficient will be biased downwards; while if the exposure positively predicts selection and the outcome negatively predicts selection (or vice versa), then the result will be biased upwards (some simple examples demonstrating this can be found in Table S12 ^25^). As an example, males are less likely to continue participating in ALSPAC, while religious attendance is likely positively associated with participation; this means that the sex coefficient could be biased upwards, compared to the true value. To explore this possibility, we performed multiple imputation to assess how the complete-case and imputed results differ for four exposures (sex, ethnicity, education and income). Overall, the patterns of results were broadly similar between the complete-case and imputed results, although in many cases – and especially for the outcome religious attendance – the magnitude of the effect size differed somewhat. For instance, the association between being male and being less likely to attend at a place of worship was stronger in the imputed data compared to the complete-case analysis, plausibly because the complete-case result was biased upwards, as described above. Full results, with additional discussion, can be found in Table S13 ^25^, along with further details on the imputation methods. These results suggest that, although selection bias may be present, it is unlikely to dramatically alter the conclusions of the complete-case analyses presented above.

Finally, we note some other limitations with this paper. First, there are many other factors not explored here which may also cause RSBB, including cognitive/psychological factors, cultural transmission/credibility-enhancing displays, and life events, among others ^9, 10, 39, 40^. The scope of this specific paper is limited to demographic and socioeconomic variables, and future work will explore associations between these other factors and RSBB in ALSPAC. Second, as we only adjusted for age and sex, many of the associations reported here may be biased by residual confounding; however, as the aim of this paper is purely descriptive and to help inform future work, we note again that the associations reported here should not be interpreted as causal estimates. A final limitation concerns the generalisability of these findings. There appears to be considerable between-study variation regarding the relationship between RSBB and sociodemographic variables – and even between the two ALSPAC generations ^14^ – which makes it difficult to know how generalisable these results are, especially across societies, generations and religions ^13, 28^. Different measures of RSBB, and different measures of sociodemographic variables, also hinder between-study comparisons, meaning it is difficult to know whether differences between studies are due to differences between the populations or differences in measurement, or both.

## Conclusions

In a cohort of young adults born in Southwest England, we find evidence for various associations between sociodemographic factors and religious/spiritual beliefs and behaviours, particularly regarding sex, ethnicity, education, income, relationship status and being a parent. Relationships were quite weak and rather variable, however, with considerable heterogeneity in results depending on the sociodemographic exposure and RSBB outcome assessed. Compared to their parents, these associations are noticeably weaker and more inconsistent, highlighting the changing nature of RSBB between generations. By describing these associations, we hope this study will be informative for future users of this ALSPAC data, and especially when thinking about potential demographic and socioeconomic confounders in future research in this cohort.

## Ethical approval and consent

Ethical approval for the study was obtained from the ALSPAC Ethics and Law Committee and the Local Research Ethics Committees. Informed consent for the use of data collected via questionnaires and clinics was obtained from participants following the recommendations of the ALSPAC Ethics and Law Committee at the time. Questionnaires were completed in the participants own home and return of the questionnaires was taken as continued consent for their data to be included in the study. Full details of the approvals obtained are available from the study website ( http://www.bristol.ac.uk/alspac/researchers/research-ethics/). Study members have the right to withdraw their consent for elements of the study or from the study entirely at any time.

## Data Availability

Please see the ALSPAC data management plan which describes the policy regarding data sharing ( http://www.bristol.ac.uk/alspac/researchers/data-access/documents/alspac-data-management-plan.pdf), which is by a system of managed open access. Data used for this submission will be made available on request to the Executive (alspac-exec@bristol.ac.uk). The datasets presented in this article are linked to ALSPAC project number B3911, please quote this project number during your application. Analysis code supporting this submission is openly-available at: https://github.com/djsmith-90/AnalysisCode_PredictorsOfRSBB_B3911. The steps below highlight how to apply for access to the data included in this study and all other ALSPAC data: 1. Please read the ALSPAC access policy ( http://www.bristol.ac.uk/media-library/sites/alspac/documents/researchers/data-access/ALSPAC_Access_Policy.pdf) which describes the process of accessing the data and samples in detail, and outlines the costs associated with doing so. 2. You may also find it useful to browse our fully searchable research proposals database ( https://proposals.epi.bristol.ac.uk/?q=proposalSummaries), which lists all research projects that have been approved since April 2011. 3. Please submit your research proposal ( https://proposals.epi.bristol.ac.uk/) for consideration by the ALSPAC Executive Committee. You will receive a response within 10 working days to advise you whether your proposal has been approved. Open Science Framework: Supplementary information supporting this submission can be found on the Open Science Framework “Demographic and socioeconomic predictors of religious/spiritual beliefs and behaviours in a prospective cohort study (ALSPAC) in Southwest England: Results from the offspring generation” project page ( https://doi.org/10.17605/OSF.IO/FY2W6) ^25^ This project contains the following extended data: “G1SocioDemoPredictorsOfRSBB_SuppInfo.pdf” (the supplementary information file) “G1SocioDemoPredictorsOfRSBB_STROBE.pdf” (the completed STROBE cohort study reporting guidelines checklist). Data are available under the terms of the Creative Commons Attribution 4.0 International license (CC-BY 4.0)
