# Supplementary material for: Demographic and socioeconomic predictors of religious/spiritual beliefs and behaviours in a prospective cohort study (ALSPAC) in Southwest England: Results from the offspring generation
Source: Wellcome Open Res. 2024 Jun 4;7:290. Originally published 2022 Dec 5. [Version 3] doi: 10.12688/wellcomeopenres.18517.3 (PMC11140305; doi:10.12688/wellcomeopenres.18517.3)
Supplement: Peer review history

# Reviewer report: Reviewer response for version 2

**DOI:** 10.21956/wellcomeopenres.22959.r71943

**Published:** 2024-05-31

**Authors:** Yingling Liu

**Affiliations:** 1 Department of Sociology, Birmingham Southern College, Birmingham, Alabama, USA

I appreciate the authors took time to read the comments and made the revisions. The article is now ready to be approved..

Is the work clearly and accurately presented and does it cite the current literature?

Partly

If applicable, is the statistical analysis and its interpretation appropriate?

Yes

Are all the source data underlying the results available to ensure full reproducibility?

Yes

Is the study design appropriate and is the work technically sound?

Yes

Are the conclusions drawn adequately supported by the results?

Yes

Are sufficient details of methods and analysis provided to allow replication by others?

Yes

Reviewer Expertise:

Health and well-being, religion and well-being, aging, older adults, inequality, life course perspective

I confirm that I have read this submission and believe that I have an appropriate level of expertise to confirm that it is of an acceptable scientific standard.

# Reviewer report: Reviewer response for version 2

**DOI:** 10.21956/wellcomeopenres.22959.r83315

**Published:** 2024-05-30

**Authors:** Nitzan Peri-Rotem

**Affiliations:** 1 Department of Sociology, University of Exeter, Exeter, England, UK

This is a well written descriptive study on the associations between demographic and socioeconomic factors and different measures of religiosity, using the ALSPAC data. The study includes some interesting findings on the offspring generation (aged around 28 years), including differences in religious measures by sex, ethnicity and socioeconomic factors.

 There are however a few shortcomings to this study:

1. The introduction is heavily focussed on health implications of religiosity or the general link between religiosity and health. However, health is not included in the analysis. It would make more sense to describe the importance of religion, not only to health, but also to other demographic and political outcomes.
2. Although the data includes information on parental religiosity, and while other parental characteristics are used as predictors of children’s religiosity, the correlation between parents and children’s religiosity is not explored in this study. This is a major limitation, as parents’ religiosity is potentially the most important predictor of children’s religiosity. It would have been useful to run a multiple regression analysis that includes parental religiosity among other socio-demographic variables to predict offspring’s religiosity. Also, it may be of interest to estimate interactions between parents’ religiosity and the sex and/or ethnicity of the offspring in relation to their current religiosity.
3. In connection to the previous point, some of the figures and tables may be redundant. For example, it is unclear how the analysis shown in figure 1 contributes to the study objectives?
4. The authors mention that information on RSBB is only available for 30% of the ALSPAC offspring generation, but it is not clear why there is such a high proportion of missing data. Is it due to attrition or refusal of participants in the study?
5. In the strengths and limitations section there is a comparison of the distribution of religious affiliation among the ALSPAC offspring to its distribution in the population census. However, the ALSPAC offspring includes a specific birth cohort of people around the age of 28, which is not comparable to the entire population. Instead, the comparison should be to the religious distribution among people around the same age.

Is the work clearly and accurately presented and does it cite the current literature?

Yes

If applicable, is the statistical analysis and its interpretation appropriate?

Partly

Are all the source data underlying the results available to ensure full reproducibility?

Yes

Is the study design appropriate and is the work technically sound?

Partly

Are the conclusions drawn adequately supported by the results?

Yes

Are sufficient details of methods and analysis provided to allow replication by others?

Partly

Reviewer Expertise:

Demography; fertility; life course analysis

I confirm that I have read this submission and believe that I have an appropriate level of expertise to confirm that it is of an acceptable scientific standard, however I have significant reservations, as outlined above.

# Reviewer report: Reviewer response for version 2

**DOI:** 10.21956/wellcomeopenres.22959.r71942

**Published:** 2024-02-02

**Authors:** Anastasia Ejova

**Affiliations:** 1 School of Psychology, University of Adelaide, Adelaide, SA, Australia; 2 Department of Psychology, Macquarie University, Sydney, NSW, Australia

The authors have addressed my comments very thoroughly, and I look forward to seeing the final version of this manuscript.

Is the work clearly and accurately presented and does it cite the current literature?

Yes

If applicable, is the statistical analysis and its interpretation appropriate?

Partly

Are all the source data underlying the results available to ensure full reproducibility?

Yes

Is the study design appropriate and is the work technically sound?

Partly

Are the conclusions drawn adequately supported by the results?

Yes

Are sufficient details of methods and analysis provided to allow replication by others?

Yes

Reviewer Expertise:

religious beliefs, cultural evolution, mental health, chronic pain

I confirm that I have read this submission and believe that I have an appropriate level of expertise to confirm that it is of an acceptable scientific standard.

# Reviewer report: Reviewer response for version 1

**DOI:** 10.21956/wellcomeopenres.20534.r68984

**Published:** 2023-11-28

**Authors:** Yingling Liu

**Affiliations:** 1 Department of Sociology, Birmingham Southern College, Birmingham, Alabama, USA

This study investigated the associations between possible demographic and socioeconomic causes of religious/spiritual beliefs and behaviors in the offspring generation of the ALSPAC. The study used correlations and multinomial regressions to exam the differences in the patterns of association between the two generations. 

 The study design is sufficient to answer the research question, and the analysis as well as the interpretation are appropriate. I am impressed with the analysis and how adequate the authors address the result and relate to the research questions. I have only two comments that hopefully can make the paper better:

1. It would be helpful to state the hypothesis clearly in the paper, preferably at the end of the introduction or in the method section. It would be easier to follow if the hypothesis were presented prior to the data analysis. 
2. The front end of the paper seems slim as it needs a more thorough set up for the analysis. The review of literature and the theoretical background can be strengthened more by including the area of research on life course perspective, religious beliefs and health. Below are some articles that may be helpful ^1^^-^^3^

Is the work clearly and accurately presented and does it cite the current literature?

Partly

If applicable, is the statistical analysis and its interpretation appropriate?

Yes

Are all the source data underlying the results available to ensure full reproducibility?

Yes

Is the study design appropriate and is the work technically sound?

Yes

Are the conclusions drawn adequately supported by the results?

Yes

Are sufficient details of methods and analysis provided to allow replication by others?

Yes

Reviewer Expertise:

Health and well-being, religion and well-being, aging, older adults, inequality, life course perspective

I confirm that I have read this submission and believe that I have an appropriate level of expertise to confirm that it is of an acceptable scientific standard, however I have significant reservations, as outlined above.

## References

[1] : Does Religious Participation Predict Future Expectations About Health? Using a Life Course Framework to Test Multiple Mechanisms. *Journal of Religion and Health* .2022;61(3) : 10.1007/s10943-021-01441-0 2539-2568 10.1007/s10943-021-01441-0 34655398

[2] : Religious Attendance and Physical Health in Later Life: A Life Course Approach. *Journal of Health and Social Behavior* .2020;61(4) : 10.1177/0022146520961363 486-502 10.1177/0022146520961363 33047980

[3] : Changes in Religious Doubt and Physical and Mental Health in Emerging Adulthood. *Journal for the Scientific Study of Religion* .2021;60(2) : 10.1111/jssr.12712 332-361 10.1111/jssr.12712

# Response

**Published:** 2023-12-15

**Authors:** Daniel Smith

**Affiliations:** University of Bristol, UK

*We thank the reviewer for their positive review and their constructive comments. We have responded to the reviewer’s comments in turn below, with the reviewer’s original comments in standard font and our responses in italics.*   I have only two comments that hopefully can make the paper better: 1. It would be helpful to state the hypothesis clearly in the paper, preferably at the end of the introduction or in the method section. It would be easier to follow if the hypothesis were presented prior to the data analysis. *As this paper is purely descriptive, we are not testing specific hypotheses. Instead, we are simply describing the associations between a range of sociodemographic factors and religious/spiritual beliefs and behaviours (RSBB) in this ALSPAC offspring sample. This broader aim of our research is detailed in the final paragraph of the discussion: “In this paper, we examine whether a wide range of demographic and socioeconomic factors are associated with RSBB in the offspring from a prospective birth cohort (the Avon Longitudinal Study of Parents and Children; ALSPAC).”. *   2. The front end of the paper seems slim as it needs a more thorough set up for the analysis. The review of literature and the theoretical background can be strengthened more by including the area of research on life course perspective, religious beliefs and health. Below are some articles that may be helpful. *Thank you for this suggestion and for the articles. We have expanded the first paragraph of the  introduction to mention these life course studies and provide greater context for our analyses: “Longitudinal research taking a life course perspective has also highlighted how the timing of religious exposures across the life-span can impact health and health perceptions, providing an insight into the mechanisms by which sociocultural factors such as religion may shape health (Upenieks & Schafer, 2020; Upenieks & Liu, 2021)”.*

# Reviewer report: Reviewer response for version 1

**DOI:** 10.21956/wellcomeopenres.20534.r53826

**Published:** 2023-02-03

**Authors:** Anastasia Ejova

**Affiliations:** 1 School of Psychology, University of Adelaide, Adelaide, SA, Australia; 2 Department of Psychology, Macquarie University, Sydney, NSW, Australia

This well-written and well-presented paper sought to identify demographic and socioeconomic factors that might, through their relationship with three different markers of religiosity, mediate the relationship between religiosity and health, or confound the relationship between religiosity and health. The paper’s aims were purely descriptive, and health was not measured or included in the analysis. All demographic and socioeconomic factors were explored individually as predictors, controlling for gender and age, and also exploring the effect of the interaction between the factor in the model and gender and age. The sample consisted of 4,500 young people born in Southwest England.

 I think descriptive work with large samples outside the US is really lacking in this area, and so I read the paper with great interest. There are a number of areas for improvement I noticed, addressing which would make the paper more impactful.

1. This is easily fixable but conceptually critical: I disagree that demographic and socioeconomic factors can be described as ‘causes’ of religious beliefs and behaviour. The mechanisms involved in any causality are unknown, and so terms like ‘contributors’ and/or ‘predictors’ are more appropriate.
2. This is probably the most critical point. Any set of extant data has a configuration of variables that a subsequent study might not be able to collect in full. In a descriptive analysis, I would be more interested in how broader types (rather than study-specific indices) of demographic and socioeconomic factors predict religious beliefs and behaviour. As such, I would have liked to see a principal components analysis of the available predictors, and then a multiple regression assessing the effects of components and theoretically meaningful interactions between components. The sample size is large enough to allow for this kind of analysis.
3. It would be great to see more acknowledgement regarding the fact that the results might not generalise to countries beyond Britain, as each country has unique history of development of religious diversity.
4. It would also be great to see – in the Method and/or Discussion – comment on the extent to which the sample is representative of the UK population. The small percentage of participants of non-Christian faiths surprises me, although it might be close to census estimates.

Is the work clearly and accurately presented and does it cite the current literature?

Yes

If applicable, is the statistical analysis and its interpretation appropriate?

Partly

Are all the source data underlying the results available to ensure full reproducibility?

Yes

Is the study design appropriate and is the work technically sound?

Partly

Are the conclusions drawn adequately supported by the results?

Yes

Are sufficient details of methods and analysis provided to allow replication by others?

Yes

Reviewer Expertise:

religious beliefs, cultural evolution, mental health, chronic pain

I confirm that I have read this submission and believe that I have an appropriate level of expertise to confirm that it is of an acceptable scientific standard, however I have significant reservations, as outlined above.

# Response

**Published:** 2023-12-15

**Authors:** Daniel Smith

**Affiliations:** University of Bristol, UK

*We thank the reviewer for their positive review and their constructive comments. We also apologise for the length of time taken to respond to the reviewer’s comments; it took quite a while to find a second reviewer for our paper, and we thank this reviewer for their patience. We have responded to the reviewer’s comments in turn below, with the reviewer’s original comments in standard font and our responses in italics.*   1. This is easily fixable but conceptually critical: I disagree that demographic and socioeconomic factors can be described as ‘causes’ of religious beliefs and behaviour. The mechanisms involved in any causality are unknown, and so terms like ‘contributors’ and/or ‘predictors’ are more appropriate. *We thank the reviewer for raising this important point, but disagree that we need to change our use of such causal language. We believe that this language is justified in this instance, as the aim of this paper is to describe the relations between religious/spiritual beliefs and behaviours (RSBB) and sociodemographic factors, with the latter likely to be used as confounders in future work; for a variable to be a confounder, it has to cause the exposure and the outcome (Hernán & Robins, 2020). If a variable is merely correlated with (or ‘predicts’) RSBB, it may be a confounder (if it causes RSBB), but could also be a mediator or a collider (if it is caused by RSBB), or have no causal association if the correlation is due to an unmeasured third variable; this distinction is crucial when considering causality, as adjustment for confounders removes bias while adjustment for mediators and colliders can result in increased bias (VanderWeele et al., 2021).* *Given this, and the theoretical expectation that sociodemographic factors are likely to cause RSBB (e.g., references 9, 10, and 13-18 cited in the text) – that is, if these sociodemographic factors were to change, so would RSBB (on average) – we believe that the use of causal language when selecting sociodemographic confounders is warranted. Of course, there is the possibility that these sociodemographic factors may also be caused by RSBB (in which case they may not only be confounders, but also potential mediators or colliders), which we discuss in detail in both the introduction and discussion. We are also clear throughout the manuscript that although our choice of sociodemographic variables was based on causal considerations, the results of our analyses may not reflect causal effect estimates. We hope this provides sufficient justification for our choice of causal language when discussing the choice of potential sociodemographic variables.* *Regarding the term ‘predictors’, we would contend that ‘prediction’ is a non-causal term and is largely synonymous with ‘associated’ or ‘correlated’ (see, e.g., Hernán, 2018 and Shmueli, 2010). For instance, carrying a lighter predicts – is correlated with – whether someone smokes, but it does not cause smoking behaviour. Regarding ‘contributors’, this would appear to us to be largely synonymous with ‘cause’ (i.e., if a factor contributes to an outcome, it causes it); we would therefore prefer to keep our original causal language to ensure that our meaning is clear.*   2. This is probably the most critical point. Any set of extant data has a configuration of variables that a subsequent study might not be able to collect in full. In a descriptive analysis, I would be more interested in how broader types (rather than study-specific indices) of demographic and socioeconomic factors predict religious beliefs and behaviour. As such, I would have liked to see a principal components analysis of the available predictors, and then a multiple regression assessing the effects of components and theoretically meaningful interactions between components. The sample size is large enough to allow for this kind of analysis. *Thank you for this suggestion. While some papers do include methods such as principal components analysis (PCA) to reduce and combine socioeconomic factors, we have not adopted this approach here for a number of reasons.* *As this is an important consideration when using these data, we have included a paragraph on this in the discussion: “In this paper we have explored associations between individual aspects of SEP (education, income, housing status, etc.), rather than combine these variables together into composite measures of SEP (e.g., via principal components analysis). This focus on individual facets of SEP was chosen for numerous reasons. First, composite measures of SEP only include variation common to all SEP items, which can lose item-specific information; while the SEP items here are somewhat correlated (Figure 1), these correlations are far from perfect, meaning that these variables likely measure somewhat different aspects of SEP. Second, combining different SEP variables together overlooks the fact that different aspects of SEP can have different associations with health and other outcomes (Braveman et al., 2005; Duncan & Magnuson, 2002). For instance, in the current study higher educational attainment was associated with more frequent religious attendance (Figure 4), but higher income was associated with lower levels of religious attendance (Figure 5); these important differences would be lost if SEP was combined into a composite variable. Third, composite SEP variables ignore the causal relations between the different facets of SEP, which are important to take into consideration and can make such composite SEP variables difficult to interpret; for instance, educational attainment likely causes later income in adulthood, yet if these variables were combined together this important information would be lost. Given these considerations, we have used individual, rather than composite, SEP items throughout this paper (for similar considerations and recommendations, see Braveman et al., 2005; Duncan & Magnuson, 2002; Rehkopf et al., 2016; VanderWeele, 2022).”.* *For these reasons, when comparing between studies, comparing like with like (i.e., education with education) may be more informative than comparing principal components of SEP which comprise different socioeconomic factors. Of course, researchers can create PCAs if they wish, but that is not an approach which we would necessarily advocate for and so have not included in our paper. Additionally, while other studies may not have the full range of SEP variables explored here, many – such as education, income and employment/occupation – are key aspects of SEP which the majority studies will collect, facilitating comparisons between this and other studies.*   3. It would be great to see more acknowledgement regarding the fact that the results might not generalise to countries beyond Britain, as each country has unique history of development of religious diversity. *The generalisability section of the discussion has now been expanded based on this point and the point below: “A final limitation concerns the generalisability of these findings. There appears to be considerable between-study variation regarding the relationship between RSBB and sociodemographic variables – and even between the two ALSPAC generations ^14^ – which makes it difficult to know how generalisable these results are, especially across societies, generations and religions. For instance, previous work has demonstrated that associations between both gender and education with RSBB are highly-variable across cultures ^13, 28^ . This sample is also not representative of the broader UK population, as ALSPAC contains a greater proportion of individuals from a White ethnic background and higher SEP compared to the UK average ^20^. This ALSPAC sample is also not representative of the wider UK population in terms of religiosity; for instance, compared to 2021 UK census estimates (* *https://www.ons.gov.uk/peoplepopulationandcommunity/culturalidentity/religion/bulletins/religionenglandandwales/census2021* *) 28% of ALSPAC offspring identify as Christian (vs 46% in the census), 65% have no religious affiliation (vs 37% in the census), and 7% have a non-Christian religious affiliation (vs 11% in the census). Together, these considerations mean that caution is needed when generalising the results of this study, both to the wider UK population and beyond.”.*   4. It would also be great to see – in the Method and/or Discussion – comment on the extent to which the sample is representative of the UK population. The small percentage of participants of non-Christian faiths surprises me, although it might be close to census estimates. *This has been addressed in the point above.* *Literature cited in response to reviewer:* *Braveman, P. A., Cubbin, C., Egerter, S., Chideya, S., Marchi, K. S., Metzler, M., & Posner, S. (2005). Socioeconomic status in health research: one size does not fit all. Jama, 294(22), 2879-2888.* *Duncan, G. J., & Magnuson, K. A. (2014). Off with Hollingshead: Socioeconomic resources, parenting, and child development. In Socioeconomic status, parenting, and child development (pp. 83-106). Routledge.* *Hernán, M. A. (2018). The C-word: scientific euphemisms do not improve causal inference from observational data. American journal of public health, 108(5), 616-619.* *Hernán MA, Robins JM (2020). Causal Inference: What If. Boca Raton: Chapman & Hall/CRC.* *Rehkopf, D. H., Glymour, M. M., & Osypuk, T. L. (2016). The consistency assumption for causal inference in social epidemiology: when a rose is not a rose. Current epidemiology reports, 3(1), 63-71.* *Shmueli, G. (2010). To explain or to predict? Statistical science, 25(3), 289-310.* *VanderWeele, T. J. (2022). Constructed measures and causal inference: Towards a new model of measurement for psychosocial constructs. Epidemiology (Cambridge, Mass.), 33(1), 141.* *VanderWeele, T. J., Rothman, K. J., & Lash, T. L. (2021). Confounding and Confounders. Modern epidemiology, 263-286.*
